# High Levels of Both n-3 and n-6 Long-Chain Polyunsaturated Fatty Acids in Cord Serum Phospholipids Predict Allergy Development

**DOI:** 10.1371/journal.pone.0067920

**Published:** 2013-07-10

**Authors:** Malin Barman, Sara Johansson, Bill Hesselmar, Agnes E. Wold, Ann-Sofie Sandberg, Anna Sandin

**Affiliations:** 1 Food Science, Department of Chemical and Biological Engineering, Chalmers University of Technology, Gothenburg, Sweden; 2 Department of Paediatrics, Institute of Clinical Sciences, University of Gothenburg, Gothenburg, Sweden; 3 Department of Infectious Diseases, Institute of Biomedicine, University of Gothenburg, Gothenburg, Sweden; 4 Department of Clinical Sciences, Paediatrics, Umeå University, Umeå, Sweden; Ludwig-Maximilians-University Munich, Germany

## Abstract

**Background:**

Long-chain polyunsaturated fatty acids (LCPUFAs) reduce T-cell activation and dampen inflammation. They might thereby counteract the neonatal immune activation and hamper normal tolerance development to harmless environmental antigens. We investigated whether fatty acid composition of cord serum phospholipids affects allergy development up to age 13 years.

**Methods:**

From a population-based birth-cohort born in 1996/7 and followed until 13 years of age (n = 794), we selected cases with atopic eczema (n = 37) or respiratory allergy (n = 44), as well as non-allergic non-sensitized controls (n = 48) based on diagnosis at 13 years of age. Cord and maternal sera obtained at delivery from cases and controls were analysed for proportions of saturated, monounsaturated and polyunsaturated fatty acids among serum phospholipids.

**Results:**

The cord serum phospholipids from subject who later developed either respiratory allergy or atopic eczema had significantly higher proportions of 5/8 LCPUFA species, as well as total n-3 LCPUFA, total n-6 LCPUFA and total LCPUFA compared to cord serum phospholipids from controls who did not develop allergy (P<0.001 for all comparisons). Conversely, individuals later developing allergy had lower proportion of the monounsaturated fatty acid 18∶1n-9 as well as total MUFA (p<0.001) among cord serum phospholipids. The risk of respiratory allergy at age 13 increased linearly with the proportion of n-3 LCPUFA (P_trend_<0.001), n-6 LCPUFA (P_trend_ = 0.001), and total LCPUFA (P_trend_<0.001) and decreased linearly with the proportions of total MUFA (P_trend_ = 0.025) in cord serum phospholipids. Furthermore, Kaplan-Meier estimates of allergy development demonstrated that total LCPUFA proportion in cord serum phospholipids was significantly associated with respiratory allergy (P = 0.008) and sensitization (P = 0.002), after control for sex and parental allergy.

**Conclusion:**

A high proportion of long-chain PUFAs among cord serum phospholipids may predispose to allergy development. The mechanism is unknown, but may involve dampening of the physiologic immune activation in infancy needed for proper maturation of the infant's immune system.

## Introduction

The prevalence of allergic diseases increased drastically in industrialized countries during the 20th century. Although genetic factors play a role, migration and adoption studies point to the environment during the first year(s) of life as a major determinant for the risk of allergy development [1]. A vast reduction in exposure to microbes has occurred in the last 100 years and allergy development has been linked to a paucity of early infections [2] or an impoverished early gut microbiota [3]. These findings suggest that the infant's immune system needs to receive stimulating signals in order to mature correctly. Such maturation could be needed to develop the capacity to actively tolerize harmless environmental antigens, “allergens”.

Another observation is the link between an allergy and replacement of butter by margarines and vegetable oils in the diet [4]–[7]. Butter is rich in saturated fatty acids, while margarines and oils are rich in polyunsaturated fatty acids (PUFAs). PUFAs are immunomodulators foremost reducing T-cell proliferation [8]–[10] and interferon-gamma production [11], [12]. This is true for both n-6 PUFAs [13]–[16], found mainly in margarine and vegetable oils, and n-3 LCPUFAs [13], [17], [18], mainly found in fish. As LCPUFAs are essential in foetal development, they are actively and selectively transported across the placenta and the newborn infant has higher levels of several LCPUFAs than those found in maternal serum [19].

The objective of this study was to investigate whether levels of long-chain fatty acids in cord serum phospholipids are associated with allergy development up to age 13 years. We speculated that if LCPUFAs in the infant's blood dampen the immune activation afforded by microbial antigens during early infancy, high LCPUFA levels might hamper immune maturation and development of tolerance to innocuous antigens, leading to increased risk of allergy development. To investigate this hypothesis, we retrospectively analysed the fatty acid composition of cord sera of individuals who developed allergy during childhood (cases) or remained non-allergic (controls).

## Methods

### Birth cohort

A total of 129 cases and controls were chosen from a population-based birth-cohort targeting all 1228 children born vaginally during one year (1996–7) in the County of Jämtland in Northern Sweden [20] and followed until 13 years of age. Prospective parents were recruited at gestational week 18 when a detailed questionnaire was distributed. The children were examined regarding sensitization to common food and inhalant allergens by skin prick tests at 1 and 4 years of age and allergic symptoms were reported by the parents through questionnaires when the children were 1, 4, and 7 years old [20]. At 13 years of age, families that still lived in the region received a new symptom questionnaire. This was accepted by 841 families and returned by 834; 794 adolescents were skin prick tested. One nurse conducted all prick tests for milk, egg, fish, wheat, soy, cat, dog, horse, timothy grass, and birch using standardized extracts (ALK, Hörsholm, Denmark; 10 histamine equivalent prick units potency). Reproducibility was checked repeatedly according to international recommendations [21].

### Ethic statement

The study was conducted according to the Helsinki II Declaration (http://www.wma.net/en/30publications/10policies/b3/index.html) and was approved by the local ethical committee in Umeå, Sweden (Dnr 95–149, 09–017 M and 09–110 M). Pregnant women were recruited in their gestational week 18. Participating was voluntary and those mothers who accepted to participate in the study provided written consent forms concerning collection of cord serum. Thirteen years later, on behalf of the minors, parents of the adolescents provided a new written consent form and the adolescents themselves approved their participation orally for skin prick tests and for fatty acid analyses in stored cord serum. As participation was voluntary, adolescents were free to at any time, without any stated reason, decide not to participate in further tests and questionnaires. All potential participants who declined no further participation or otherwise did not participate were not disadvantaged in any way by their decision.

### Collection of serum samples

Cord and maternal venous serum were obtained at delivery and stored frozen at −20°C until analysed.

### Allergy diagnosis

Study subjects were selected based on the clinical diagnosis at 13 years of age and included the following three diagnostic groups:

1. Respiratory allergy, defined as fulfilling ≥1 of the following criteria; wheeze in the past year, doctor's diagnosed asthma, asthma inhalation treatment, or a positive answer to the question: “Have you had any signs of pollen allergy or allergy to furred pets during the last 12 months?” Further, at least one positive reaction in the skin prick test was required and the individual should have no other atopic diagnosis. In all, 139 adolescents fulfilled these criteria.
2. Atopic eczema, defined as pruritic, chronic or chronically relapsing non-infectious dermatitis with typical features and distribution, fulfilling three of the main criteria suggested by Hanifin and Rajka [22]. Sensitisation was not required, but the individual should display no other allergic disease manifestation. In all, 79 adolescents fulfilled these criteria.
3. Non-allergic controls, defined as having neither allergic symptoms, nor any positive reaction in the skin prick test at 13 years of age (n = 332).

### Selecting subjects

From the three groups, we aimed to select approximately 50 subjects per group for fatty acid analysis of stored cord serum samples. In the group with atopic eczema, however, only 40 subjects had sufficient amounts of sera stored for fatty acid analysis; these were all included, but due to need for reanalysis etc., 37 individuals remained in this group. For respiratory allergy and non-allergic controls, we selected 50 individuals at random and the final groups included 44 cases with respiratory allergy and 48 non-allergic, non-sensitized controls. The selected subjects were not matched on any factors, but a post-hoc analysis was done to test for skewness in the selection process. As seen in Table 1, there were no significant differences between the randomly chosen subjects and the diagnostic groups from which they derived.

**Table 1:** Characteristics of the individuals in the selected study groups, the clinical groups from which they were selected and the cohort from which they derived.

	Respiratory allergy†		Atopic eczema*		Non-allergic		All participants in cohort at 13 years of age
	Selected	Whole group	Selected	Whole group	Selected	Whole group	
	(N = 44)	(N = 130)	(N = 37)	(N = 79)	(N = 48)	(N = 332)	(N = 834)
Female sex	33	33	70	70	62	56	50
Older siblings^¤^	59	65	59	58	65	60	62
Exclusively breast-fed at 4 months^¤^	70	73	74	78	72	71	72
Maternal allergy^ §^	61	55	49	52	31	33	43
Asthma	25	17	21	23	4	6	14
Rhinitis	43	33	21	24	12	11	20
Eczema	32	32	35	38	23	23	28
Paternal allergy^§^	51	48	35	37	31	32	36
Asthma	16	16	8	8	10	11	12
Rhinitis	40	32	16	19	17	15	20
Eczema	21	19	24	25	6	16	18
Cat during 1^st^ year of life^¥^	14	12	24	23	23	19	19
Dog during 1^st^ year of life^¥^	18	16	30	29	21	30	28
Urban residence^ ¥^	69	62	67	65	61	56	58

† Fulfilling at least one of the following criteria; wheeze in the past year, asthma inhalation treatment, or an affirmative answer to the question: “Have you had any signs of pollen allergy or allergy to furred pets during the last 12 months?”, diagnosed at 13 years of age. ^*^Defined as a pruritic, chronic or chronically relapsing non-infectious dermatitis with typical features and distribution and fulfilling at least three of the main criteria defined by Hanifin and Rajka [22], diagnosed at 13 years of age. ^¤^Response to questionnaire distributed at 1 year of age. ^§^Defined as an affirmative answer to the question “Has the child's mother/father ever had asthma, hay fever or eczema?” answered at 13 years of age. ^¥^Answered at 4 years of age.

### Maternal consumption of fish during pregnancy

The questionnaire distributed to the pregnant mother at gestational week 18 contained a question about how often they consumed fish. A complete answer was received from 79 of the 129 mothers of the investigated subjects. Based on their responses, the mothers were divided into three groups: those eating fish less than twice a month (n = 18), those eating fish approximately twice a month (n = 34) and those eating fish more than twice a month (n = 27).

### Analysis of serum phospholipids

The phospholipid fraction of cord and maternal sera obtained at delivery were extracted and methylated by direct trans-esterification [23]. Fat was extracted with chloroform: methanol (1∶2) and 0.5% NaCl-solution [24]. Phospholipids were obtained by separation on amino propyl solid phase extraction columns (Isolute NH2, 6 ml, 500 mg, IST, Mid Glamorgon, UK) [25] and converted to methyl esters [23], during overnight incubation. The methyl esters were then extracted with petroleum ether. After evaporation, the phospholipids were dissolved in isooctane and separated by gas chromatography (Hewlett Packard 5890, Waldbronn, Germany). Detection was done by flame ionization and the Borwin software (Le Fontanil, France) was used for evaluation. The samples were separated by gas chromatography on two different columns. First, the 129 samples were separated on a HP Ultra 1 (50 m ×0.32 mm ×0.52 µm d_F_) silicon column (J&W Scientific, California) suitable for separation of the 20–22 carbon atoms long fatty acids. The proportion of each of these fatty acids was calculated as per cent of the total amounts of fatty acids (16–22 carbon atoms long) based on peak area. Where sufficient materials remained (85 samples), we also performed separation of 16–18 carbon long fatty acids on a DB-WAX (30 m ×0.25 mm ×0.25 µm d_F_) column (J&W Scientific, California). The proportions of these fatty acids were also expressed as percentage of total fatty acids, i.e. all fatty acids 16–22 carbon atoms long; the total amount of fatty acids is not dependent on complete resolution between all the peaks in the chromatograms and could, thus, be estimated from either of the columns.

### Statistical analysis

Chi-tests were used for the background analysis to test for differences between qualitative variables between groups, and for trend analyses. Differences in fatty acid proportions were compared between clinical groups using Mann–Whitney U test. Spearman's rank correlation was used to test the relation between fatty acid proportions in maternal and cord serum phospholipids, and between maternal fish intake and n-3 LCPUFA content in maternal and cord serum phospholipids. IBM SPSS Statistics version 19 (IBM Corporation, New York) was used.

Kaplan–Meier curves were calculated (IBM SPSS Statistics) to follow allergy development over time in subjects grouped according to cord LCPUFA levels. Cox regression was used to control for confounders.

To identify confounders, we performed partial least square (PLS), a regression variety of principal component analysis using Unscrambler® X version 10.2 (CAMO Software AS, Oslo). The advantage with PLS is that the variables need neither to be normally distributed, nor independent from one another. The clinical diagnosis consisted the response (Y) variable to which total LCPUFA proportion in cord serum phospholipids and potential confounders (sex, parental history of allergy, pregnancy length, maternal proportions of total LCPUFAs in serum at delivery, breastfeeding, older siblings and pets) were related. Confounders found to be significant in the PLS regression model were then used in the Cox-regressions.

## Results

Adolescents who either had respiratory allergy (n = 44), or atopic eczema (n = 37) or were non-allergic at 13 years of age (n = 48) were selected from a population-based birth-cohort. The characteristics of the diagnostic groups, as well as the individuals selected from each group, are shown in Table 1. Parental allergy was more common among allergic individuals, while pet-keeping during the 1^st^ year of life was less common in adolescents with respiratory allergy, compared to non-allergic ones (Table 1). This is in accordance with our previous reports from this cohort [20]. As seen from the table, selected adolescents differed little from the groups from which they derived, suggesting that no major skewness was imposed when we selected individuals for fatty acid analysis of cord sera.

### Phospholipid composition in cord serum of individuals with respiratory allergy or atopic eczema at 13 years of age, compared to non-allergic controls

Phospholipids were extracted from stored cord serum samples of individuals representing the three diagnostic groups (respiratory allergy, atopic eczema and non-allergic, respectively) and the proportions of long-chain polyunsaturated fatty acids (LCPUFA), monounsaturated and saturated fatty acids in these phospholipids were analysed. The results are shown in Table 2. Cord serum phospholipids from individuals who had developed allergy by age 13 had higher proportions of 5/8 of the examined LCPUFAs compared to individuals who were non-allergic by age 13. This included the n-3 PUFAs docosapentaenoic acid (DPA) and docosahexaenoic acid (DHA), as well as the n-6 PUFAs arachidonic acid (AA), docosadienoic acid (22∶4 n-6) and 20∶2 n-6 (Table 2). In accordance, the proportion of total n-3 LCPUFA, total n-6 LCPUFA or total LCPUFA were all higher in cord sera of individuals who had respiratory allergy (p<0.001 for all comparisons) or atopic eczema (p<0.001 for all comparisons), compared to those who were non-allergic. Cord serum from individuals who remained non-allergic at 13 years of age instead contained higher proportions of the saturated fatty acid 20∶0 and the monounsaturated fatty acid oleic acid (18∶1 n-9). They also had a higher proportion of total MUFA than individuals with respiratory allergy (p<0.001) or atopic eczema (p<0.001).

**Table 2:** Proportions of long chain fatty acids in cord serum phospholipids (% of cord serum phospholipids, mean±SEM).

Proportions of 20–22 carbon long fatty acids											
		n-3 LCPUFA			n-6 LCPUFA					SFA	Ratio
	n	20:5 n-3	22:5 n-3	22:6 n-3	20:2 n-6	20:3 n-6	20:4 n-6	22:4 n-6	22:5 n-6	20:0	Total n-6
		EPA	DPA	DHA		DHGLA	AA				/ Total n-3
Respiratory allergy	44	0.30±0.03	0.49±0.03	5.1±0.21	0.39±0.01	5.7±0.11	13±0.29	0.62±0.02	0.47±0.03	1.1±0.07	3.7±0.13
Atopic eczema	37	0.30±0.02	0.41±0.02	4.5±0.18	0.41±0.01	5.5±0.13	13±0.27	0.57±0.02	0.42±0.02	1.1±0.10	3.9±0.14
Non-allergic	48	0.26±0.02	0.32±0.02	3.6±0.15	0.36±0.01	5.4±0.14	11±0.25	0.48±0.02	0.41±0.02	2.0±0.19	4.6±0.17
p (resp vs ctrl)			<0.001	<0.001	<0.05		<0.001	<0.001		*<0.01*	*<0.001*
p (ecz vs ctrl)			<0.01	<0.001	<0.05		<0.001	<0.001		*<0.001*	*<0.05*

Proportions of 16–18 carbon long fatty acids											
		n-6 PUFA	MUFA			SFA					
	n	18:2 n-6	16:1 n-7	18:1 n-7	18:1 n-9	16:0	18:0				
		LA									
Respiratory allergy	27	7.9±0.20	1.0±0.06	3.2±0.16	10±0.53	32±0.23	16±0.22				
Atopic eczema	30	8.0±0.20	1.0±0.07	3.4±0.08	10±0.32	33±0.22	16±0.26				
Non-allergic	28	8.0±0.27	1.3±0.08	3.4±0.08	12±0.41	32±0.42	16±0.31				
p (resp vs ctrl)		*<0.05*	*<0.05*		*<0.001*						
p (ecz vs ctrl)					*<0.001*						

Phospholipids were extracted from cord serum and analysed by gas chromatography. Fatty acids of chain length 20–22 carbon atoms were analysed in all samples, while 16–18 carbon atom long fatty acids were assayed in samples of sufficient remaining quantity. Adolescents were assigned to any of the three diagnostic groups based on clinical examination at 13 years of age. Adolescents with atopic eczema had no other allergic manifestation; the same was true of adolescents with respiratory allergy. Non-allergic adolescents had no symptoms and no skin-prick test positivity to a range of common aeroallergens and food allergens (see Methods). P values given in italics signify higher levels of the fatty acid species in the infants who did not develop allergy.

N-3 and n-6 PUFAs may have both similar and unique effects on early immune development. The ratio of total n-6 over total n-3 LCPUFA in cord serum phospholipids was calculated for each individual and compared between the groups. Non-allergic individuals had a higher n-6/n-3 ratio than both individuals with respiratory allergy and atopic eczema (Table 2). This suggested that, although both n-3 and n-6 PUFAs were associated with allergy development, n-3 LCPUFAs had the stronger effect.

### Cord serum LCPUFA in relation to maternal allergy

**Figure 1:**
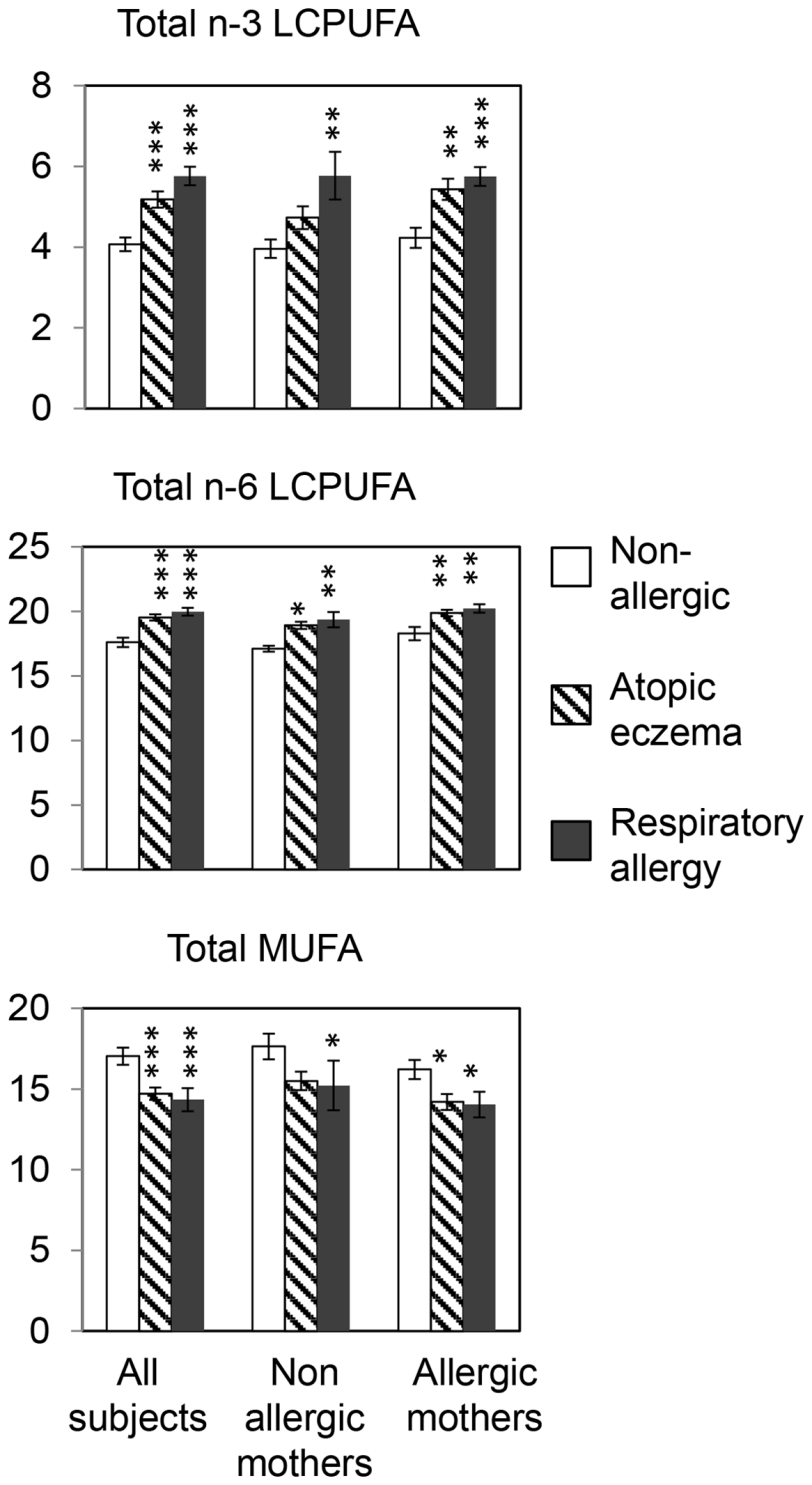
Cord serum phospholipid proportions of n-3 LCPUFA, n-6 LCPUFA and MUFA and allergy at 13 years. The figure shows the proportion of (a) n-3 long-chain polyunsaturated fatty acids (LCPUFAs), (b) n-6 LCPUFAs, and (c) total MUFAs in cord serum phospholipids in the three groups, respiratory allergy (filled), atopic eczema (hatched) or non-allergic (unfilled), for all subjects (n = 129, left), subjects born to non-allergic mothers (n = 54, middle) as wells as subjects born to allergic mothers (n = 75, right). Mann-Whitney U test were used to test for significant differences between the allergic groups and the non-allergic group, *P<0.05, **P<0.01, ***P<0.001.

As allergic infants tend to be born to allergic mothers, and since LCPUFAs in cord blood derive from the mother, maternal allergy could be a confounding factor affecting both allergy and LCPUFA levels in the offspring. We therefore split the individuals according to maternal allergy and examined the relation between LCPUFA and allergy both in children of non-allergic mothers (n = 54) and allergic mothers (n = 75). Figure 1 shows that, irrespective of allergy in the mother, individuals who were allergic by age 13 had higher proportion of n-3 LCPUFA (Fig. 1a) as well as n-6 LCPUFA (Fig. 1b) in cord serum phospholipids. Conversely, individuals who developed allergy had lower proportions of MUFA than those who remained non-allergic, irrespective of allergy in the mother (Fig 1c).

### Dose-response relation between cord LCPUFA and risk of being allergic at 13 years of age

**Figure 2:**
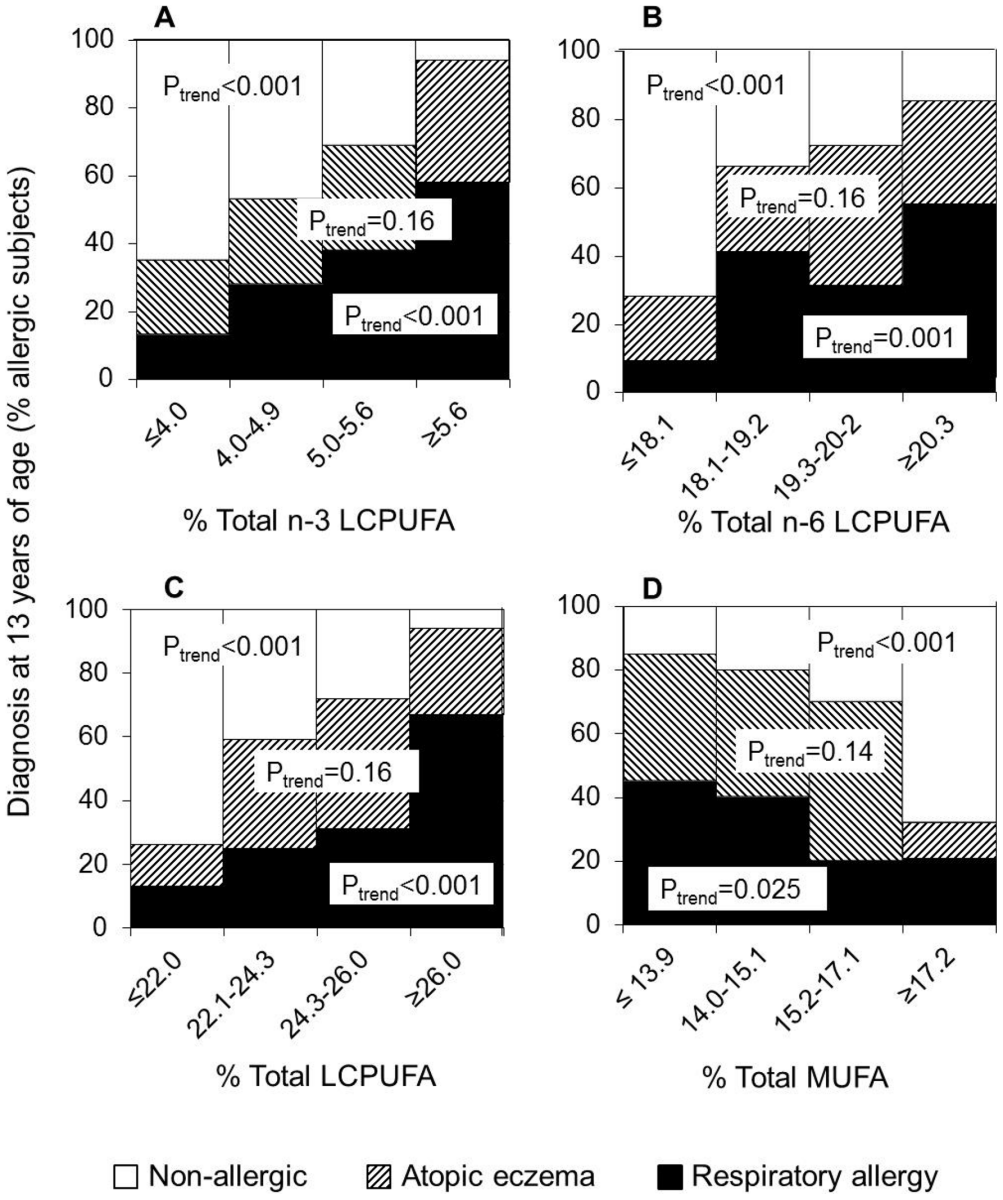
Developing different allergic manifestations as a function of fatty acid proportions in cord serum phospholipids. The 129 investigated individuals were divided into quartiles according to their proportions of(a) n-3 long-chain polyunsaturated fatty acids (LCPUFAs), (b) n-6 LCPUFAs, (c) total LCPUFAs and (d) total MUFAs in cord serum phospholipids and the proportion within each quartile that had respiratory allergy (filled), atopic eczema (hatched) or were non-allergic (unfilled) is depicted. P value for trend analysis (P_trend_) is shown for each allergic manifestation.

A dose-response between cord serum phospholipid LCPUFA proportions and the risk of being allergic by age 13 would strengthen the assumption that high proportions of LCPUFA in the cord serum would be causally related to allergy development. We stratified the 129 subjects into quartiles according to their proportions of n-3 LCPUFA, n-6 LCPUFA, total LCPUFA, and MUFA, in cord serum phospholipids. For each quartile, we determined the proportion of subjects who had respiratory allergy, atopic eczema, or were non-allergic at 13 years of age. As seen in Figure 2a, there was a strong positive linear trend between the proportion of cord serum phospholipids of the n-3 LCPUFA type and the risk of having respiratory allergy at 13 years of age (p<0.001). Conversely, there was an equally strong negative relation between n-3 LCPUFA proportion among cord serum phospholipids and being non-allergic at 13 years of age (p<0.001, Fig. 2a). Similarly, the proportion of n-6 LCPUFA in cord serum phospholipids was also linearly and positively related to having respiratory allergy at 13 years of age, and inversely related to being non-allergic at that age (Fig 2b), the same was true of the proportion of LCPUFA (sum of n-3 and n-6, Fig. 2c). Conversely, the proportion of MUFA among cord serum phospholipids, were negatively related to risk of having respiratory allergy by age 13, but positively related to being non-allergic by that age (Fig 2d). Atopic eczema at 13 years of age was not significantly dose-dependently related to either the proportions of LCPUFA or MUFA in cord serum (Fig 2a–d).

### Development of sensitization and allergy with age in relation to cord LCPUFA

**Figure 3:**
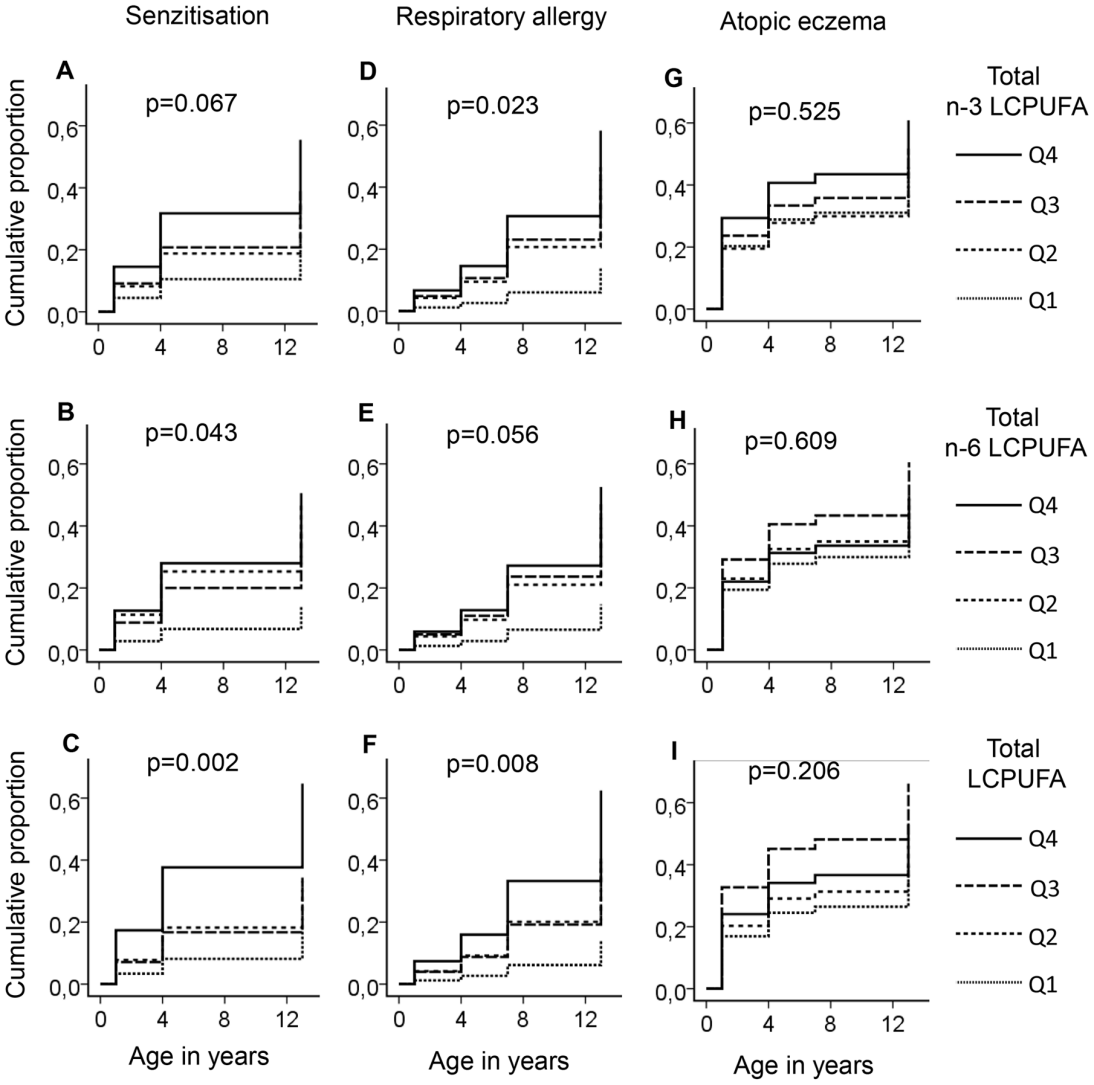
Cumulative proportion of allergy and sensitization in relation to LCPUFA proportions in cord serum phospholipids. The 129 investigated individuals were divided into quartiles (Q1–Q4) according to their proportions of total n-3 (a–c), total n-6 (d–f), and total long-chain PUFAs (g–i) among cord serum phospholipids, Q1 representing the lowest and Q4 the highest proportion. Allergy was diagnosed at 1, 4, 7 and 13 years of age and sensitization to a panel of 10 allergens was measured by skin prick test at 1, 4 and 13 years of age. Cox regression was performed and P values were adjusted for the following confounders: sex, and parental history of allergy.

Allergy develops gradually over the years, i.e. early manifestations, such as eczema and sensitization to food and airborne allergens, may progress to respiratory allergy, a phenomenon termed “the atopic march” [26]. Cord blood fatty acid composition reflects the environment in which the infantile immune system develops and should, hence, affect the early phases of the atopic march. As the subjects in the birth-cohort had been examined regarding sensitization to common allergens by skin prick tests at 1, 4, and 13 years of age, and regarding allergy symptoms by questionnaires at 1, 4, 7, and 13 years of age, we could follow sensitization and allergy development over time in individuals differing in cord serum fatty acid composition. Kaplan-Meier analyses were performed after stratification of the subjects into quartiles according to their proportions of n-3 LCPUFA, n-6 LCPUFA and total LCPUFA, respectively. As seen in Figure 3, sensitization was more prevalent from one year onwards in the individuals with the highest proportion of LCPUFA compared to those with the lowest proportion as shown for n-3, n-6 or total LCPUFA (Fig 3a–c). Along the same lines, respiratory allergy developed successively during childhood and at a higher rate in individuals who had the highest proportion of n-3 LCPUFA (Fig 3d), n-6 LCPUFA (Fig 3e), or total LCPUFA (Fig. 3f) in cord serum. Kaplan-Meier analysis failed to reveal a significant relation between LCPUFA proportion in cord serum and development of atopic eczema (Fig. 3g–i).

Cox regression analyses were used to control for confounders. We used the multivariate method PLS to identify confounders for each of the clinical entities. Figure S1, shows the result of the PLS regression model with respiratory allergy as  = y and total LCPUFA, as well as potential confounders, as X variables. Confounders that were found significant in this model were; sex (p<0.001), maternal allergy (p = 0.029) and paternal allergy (p = 0.048). Breastfeeding for at least 4 months (p = 0.332), having pets during the first year of life (p = 0.251), pregnancy length (p = 0.232), maternal levels of total LCPUFAs (p = 0.075) or having older siblings (p = 0.998) were not significant (Fig. S1). Based on this information, the P values in Fig. 3 were all adjusted for sex and parental history of allergy using Cox regression. Thus, Kaplan-Meier curves with Cox regression to control for significant confounders revealed that development of sensitization to common environmental allergens and respiratory allergy over the first 13 years of life was strongly significantly positively related to the proportion of LCPUFA in cord serum phospholipids, while no significant association could be seen between cord LCPUFA proportions and development of atopic eczema (Fig. 3).

### Contribution of maternal fish intake to serum LCPUFA in mother and infant

**Figure 4:**
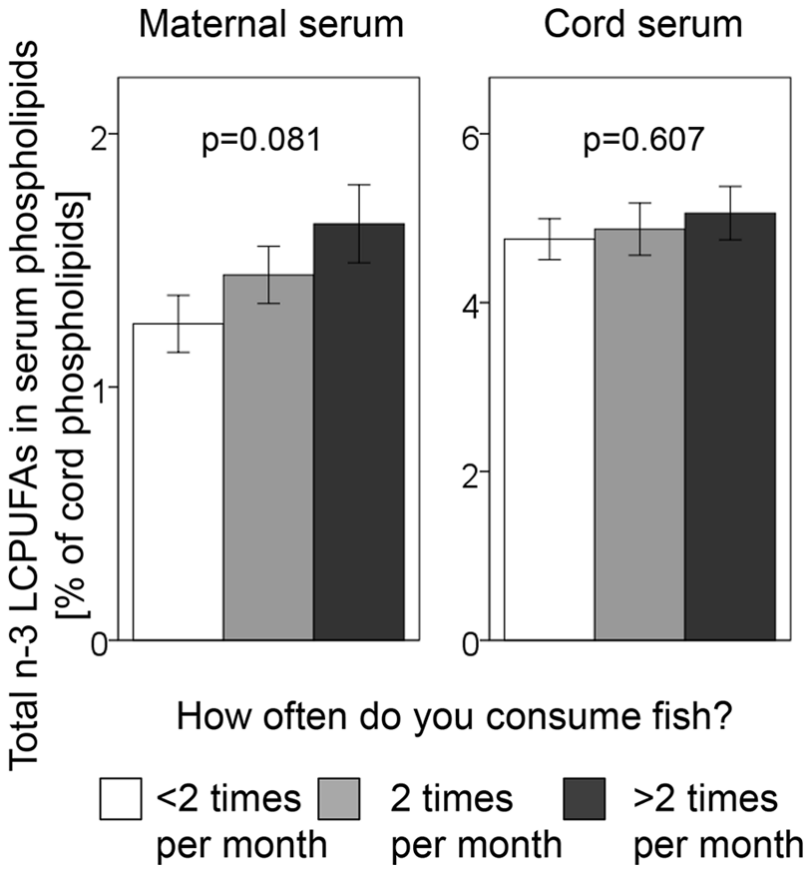
Correlation between fish intake in pregnancy and n-3 LCPUFA in maternal and cord serum phospholipids at birth. At gestational week 18 the mothers were asked to specify how often they consumed fish. These answers were then converted to intake frequencies in times per month and three groups were formed; one group eating fish less than twice a month (white bars), one group eating fish twice a month (light grey bars) and one group eating fish more than twice a month (dark grey bars). This was then correlated to the levels of total n-3 LCPUFA in maternal and cord serum phospholipids at birth.

As the newborn infant derives its fatty acids from the maternal circulation, maternal intake of food rich in certain LCPUFAs could be reflected in the cord serum of the newborn infant. Unfortunately, no diet registration was included in the study protocol. However, in mid-pregnancy, the mothers were asked about how often they consumed fish, the reason being to detect whether pregnant mothers avoided food that was, by that time, considered “allergenic”. Fish and other seafoods are the principal source of n-3 LCPUFA and Figure 4 shows the relation between the proportion of such LCPUFAs in maternal serum and the frequency of fish consumption. Although non-significant, there was a positive trend relating the pregnant woman's proportion of n-3 LCPUFA in serum phospholipids to her intake of fish. The cord serum of the newborn infants had much higher proportion of n-3 LCPUFA in the phospholipids compared to the serum of the mother, i.e. at least three times higher (Fig. 4). Furthermore, the proportion of n-3 LCPUFA in the infant's cord serum phospholipids was practically unrelated to maternal fish intake (Fig. 4). Both these observations are compatible with the active pumping of LCPUFAs across the placenta from the maternal circulation against a concentration gradient [19]. The active transport could explain the lack of significant correlations between individual species of LCPUFAs in maternal serum and cord serum phospholipids; only one LCPUFA species showed significant correlation (22∶5 n-3: Rho = 0.30, P = 0.04). In contrast, saturated fatty acids and MUFAs are thought mainly to reach the foetal circulation by passive diffusion [27]. Accordingly, we found moderate correlations between maternal and cord serum phospholipid levels of 18∶0 (Rho = 0.39, P = 0.04), and 18∶1 n-7 (Rho = 0.46, P = 0.01). Nevertheless, the magnitudes of the correlation coefficients demonstrate that maternal serum fatty acid levels are not the major determinant of cord serum phospholipid fatty acid pattern.

## Discussion

The present study demonstrates a strong and significant positive association between high proportions of long-chain PUFAs in the phospholipid fraction of cord serum and development of sensitization and respiratory allergy during the first 13 years of life. Both n-3 and n-6 LCPUFA proportions were positively associated with allergy development, while the proportion of monounsaturated fatty acids, MUFA, was inversely related to sensitization and allergy development. The effect persisted after controlling for potential confounders, such as parental allergy and sex, and both n-3 and n-6 LCPUFA proportions dose–dependently increased the risk for developing respiratory allergy. The strongest predictor for development of sensitization and respiratory allergy was the proportion of total LCPUFA in cord serum phospholipids, i.e. the sum of LCPUFAs of the n-3 and n-6 series. However, the fact that the ratio of n-6 over n-3 LCPUFA in cord serum phospholipids was higher in individuals who were non-allergic by age 13 than in those who were allergic by that age, suggests that n-3 LCPUFAs might, if anything, be more prone to support allergy development.

The milieu that surrounds the immune system may modulate immune responsiveness. Lymph nodes are embedded in perinodal adipose tissue whose fatty acid composition might impact on the local immune function [28]. As most data point to a crucial importance of the very early period of life as decisive for immune modulation and, hence, allergy development, we consider it relevant to examine the fatty acid composition of cord serum as to represent the milieu in which the infant immune system encounters the first immunostimulatory events. After birth the foetus is exposed to exogenous microbes and the immature immune system is activated. Many observations, summarized as the hygiene hypothesis [29], [30] suggest that early microbial exposure protects against allergy development. Thus, poverty [31], large families [29], early day care [32], exposure to farm animals [33] and pets [34], have all been linked to reduced risk of allergy development. The same is true of exposure to orofaecal microbes [35], [36], to a gut microbiota containing a wide variety of different bacterial types [3], [37] and to parents' salivary microbes [38]. The fatty acid milieu in which this immune stimulation takes place may affect the outcome of this stimulation.

The immune modulatory effects of PUFAs have been studied extensively. Both n-3 and n-6 PUFAs and their metabolites are immunosuppressive in that they reduce T-cell activation and signalling [8]–[10], [13], [17], [18] as well as production of interferon-gamma by T-cells [11], [12]. n-3 LCPUFAs often show somewhat stronger effect in dampening T-lymphocyte proliferation. This is in accordance with our findings that, although both n-6 and n-3 LCPUFAs were positively associated with allergy development, the effect seemed particularly pronounced for the n-3 LCPUFAs. In the presence of high-levels of LCPUFA in serum and body fluids, T-cell activation and proliferation are likely to be blunted and this may hamper the immune maturation needed to develop physiologic tolerance to harmless environmental antigens, “allergens”. Notably, LCPUFAs foremost target Th1 cells [39]–[42], which are activated by microbial exposure [43], while Th2 cells, that are central in IgE-mediated allergy, are not suppressed by either n-3 or n-6 PUFAs to the same extent [12]. Accordingly, high levels of either n-3 EPA or n-6 AA in cord serum were shown to attenuate immunologic responses of cord blood cells to *in vitro* stimulation, particularly allergen-stimulated lymphocyte proliferation and IFN-γ production [44].

The proportion of total monounsaturated fatty acids (MUFAs) correlated negatively with allergy development in a dose-response fashion. The proportion of total MUFA also correlated negatively with the proportion of total n-3 LCPUFA and total n-6 LCPUFA in cord serum phospholipids. We can therefore not distinguish whether LCPUFAs enhance allergy development, or whether MUFAs exert a protective effect. Regarding effects on the immune system, most focus has been on PUFAs, while MUFAs have been little studied. However, according to current understanding, MUFAs have no dramatic effects on inflammation or immune function (as reviewed by Galli and Calder [45] and Yaqoob [46]).

We are aware of only two prior studies that have examined the relationship between cord serum fatty acid pattern and subsequent sensitization or allergy development [47], [48]; both found no significant associations, although Duchén *et al*. [48] found higher proportions of 22∶4 n-6 and 22∶5 n-6 in serum collected at three months old atopic infants compared with non-atopic infants. The lack of significant findings in these two studies might be related to the relatively short follow-up times (18 months [48] and 3 years [47]). Yu and Björkstén [49] found higher levels of arachidonic acid, eicosapentaenoic acid (EPA), DHA, 22∶4 n-3, and total n-3 PUFAs in cord serum of infants of allergic mothers compared with infants of non-allergic mothers; the same findings were made by us. MUFA levels in cord blood in relation to allergy have, to our knowledge, not been reported.

A few randomized trials have been conducted in which pregnant women have been given n-3 LCPUFA with the purpose of reducing allergy development in their offspring [50]–[54]. Three of these studies have too short observation time to draw any firm conclusions regarding allergy development. Of note, they have also not included any group not receiving any PUFA. Dunstan *et al*. [50] gave 52 pregnant women 4 gram fish oil per day from gestational week 20 until delivery, while the control group (n = 46) received the same amount of olive oil. No significant effect of fish oil supplementation was seen for clinical allergy at one year of age. Similarly, Furuhjelm *et al*. published two follow-ups [51], [52] on a randomized control trial were pregnant and lactating women received either fish oil (n = 52) or n-6 PUFA rich soy oil (n = 65) from gestational week 25 until 3–4 month postpartum. No difference in the prevalence of allergic symptoms was found between the intervention groups at one year of age. Palmer *et al*. [54] gave pregnant women fish oil or vegetable oil capsules containing considerable amount of n-6 PUFA from gestation week 21 until birth. No effects on clinical allergy were seen at one year of age. Only one study has allowed sufficient time for adequate diagnosis of allergic disease; Olsen et**al. [53] gave pregnant women either fish oil, olive oil, or no oil capsules from gestational week 30 until birth. Asthma and eczema, as well as total allergy were assessed at 16 years of age. The olive oil group had the highest incidence of allergy. Both the fish oil and no oil groups had similar low allergy incidence at 16 years of age, albeit it was actually lowest in the no-oil group. Hence, compared to no oil supplementation, fish oil supplementation during pregnancy did not reduce allergy in the offspring.

The active pumping of PUFAs, particularly DHA, across the placenta to the foetus was clearly evident in our study from the fact that while n-3 PUFAs constituted 1–2% of maternal phospholipids, they made up 4–6% of the cord phospholipids. Furthermore, for most of the investigated fatty acids, we found no correlation between the levels in maternal and infant sera. Among the LCPUFA species, only one (22∶5 n-3) showed a modest correlation (0.30). In agreement with the assumption that saturated fatty acids and MUFAs mainly diffuse passively into the foetal circulation [27], we observed moderate correlations between maternal and cord serum phospholipid levels of 18∶0 and 18∶1 n-7 (0.39 and 0.46, respectively). Thus, the efficiency by which PUFAs are transported from the mother to the foetus across the placenta may be more important for the foetal serum levels than the maternal levels of LCPUFAs.

Our data are supported by epidemiological studies that have linked an increased risk of allergy with consumption of margarine, while consumption of butter is protective; margarine in rich in PUFAs (mainly n-6 n-6 PUFAs), while butter is rich in saturated fats [4]–[7]. Early introduction of fish into the infant's diet protects against development of eczema, as shown in several studies [55]–[58]. Although this has been interpreted as reflecting a beneficial effect of n-3 LCPUFA on allergy development, fish contains an array of other bioactive components that could confer protection against eczema in early childhood. Also, with one single exception [55], the beneficial effect of early fish introduction has not been linked specifically to fatty fish [56]–[58].

It should be pointed out that our study only examines the relation between the risk of allergy development and the fatty acid composition at birth, before activation of the immune system in response to external stimuli. Once the infant is born and exposed to exogenous antigens, PUFAs may be consumed in the body. Hence, in an animal model of allergen-induced sensitisation and respiratory inflammation, both n-3 and n-6 LCPUFAs in the serum were rapidly reduced, suggesting that they are consumed during immune activation and inflammation [59]. This may explain why already allergic individuals may have reduced proportions of n-3 LCPUFAs [60].

A limitation of this study is that it is not population based; we did not analyse fatty acids in all available subjects as these analyses are quite costly and time-consuming. Instead, we randomly selected individuals from a population-based cohort encompassing all vaginally delivered infants born in one hospital during one year. Selection was based on the diagnosis at 13 years of age and the selected individuals did not differ with respect to any measured variables from the rest of the individuals in the same clinical group. The long follow-up time with repeated skin prick tests and questionnaires allowed us to study only clearly defined clinical cases. Interestingly, high proportions of LCPUFAs were strongly linked to development of sensitization and respiratory allergy, while there was much less strong evidence of an effect on the other disease phenotype, i.e. having atopic eczema, but no other allergic disease manifestation at 13 years of age. This is, admittedly, a relatively rare phenotype, since atopic eczema tends to wane and give way to respiratory allergy. Clearly, atopic eczema that persists into adolescence without concomitant other allergic manifestations, is not a prototype Th2-driven reaction. Accordingly, only a minority of the eczematous individuals were sensitized against common environmental allergens by 13 years of age. In eczematous lesions, Th2 cells dominate early, but are largely replaced by Th1 cells during the chronic phase and a substantial proportion of CD8+ T-cells are also seen in the infiltrate [61]. Furthermore, genetic predisposition may play a greater role in this disease phenotype than in sensitization and respiratory allergy.

In summary, our study demonstrates a positive dose–response relationship between levels of both n-3 and n-6 PUFAs in cord serum and subsequent development of sensitization and respiratory allergy. A tentative explanation could be that n-3 and n-6 LCPUFAs counteract T-cell activation in response to microbial exposure and thereby delay the maturation of the infant's immune system that is needed to develop tolerance to innocuous environmental antigens.

## Supporting Information

Figure S1 **PLS regression on possible confounding variables.** In a PLS regression model respiratory allergy was used as the response variable (Y) and the total LCPUFA proportion in cord serum phospholipids together with confounders were used as X-variables. The figure shows the weighted regression coefficients. The striped bars are significant and the solid bars are non-significant. The error bars represent standard deviation for the weighted regression coefficients.
(TIF)
