# Supplementary figures and images for: High Levels of Both n-3 and n-6 Long-Chain Polyunsaturated Fatty Acids in Cord Serum Phospholipids Predict Allergy Development
Source: PLoS One. 2013 Jul 10;8(7):e67920. doi: 10.1371/journal.pone.0067920 (PMC3707846; doi:10.1371/journal.pone.0067920)

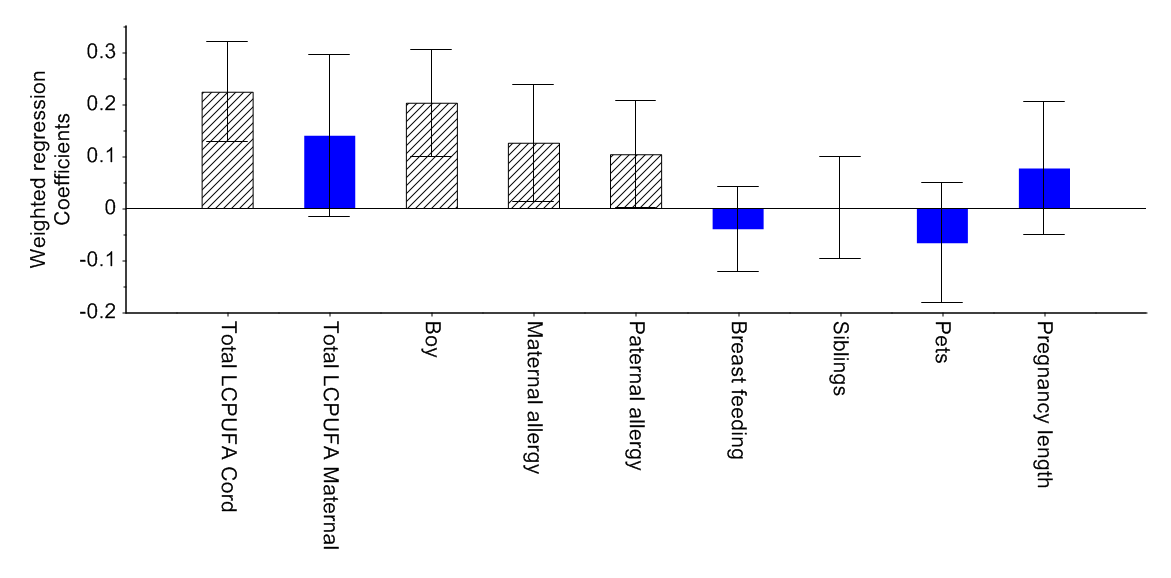

Supplement: Figure S1 — PLS regression on possible confounding variables. In a PLS regression model respiratory allergy was used as the response variable (Y) and the total LCPUFA proportion in cord serum phospholipids together with confounders were used as X-variables. The figure shows the weighted regression coefficients. The striped bars are significant and the solid bars are non-significant. The error bars represent standard deviation for the weighted regression coefficients. (TIF) [file pone.0067920.s001.tif]
